# Continuous detrimental activity of intra-articular fibrous scar tissue in correlation with posttraumatic ankle osteoarthritis

**DOI:** 10.1038/s41598-023-47498-7

**Published:** 2023-11-16

**Authors:** Nhat Tien Tran, Sang-Hyeon Jeon, Young Jae Moon, Kwang-Bok Lee

**Affiliations:** 1 Department of Surgery, University of Medicine and Pharmacy, Hue University, Hue, Vietnam; 2 Department of Orthopaedic Surgery, Research Institute of Clinical Medicine of Jeonbuk National University-Biomedical Research Institute of Jeonbuk National University Hospital, Jeonbuk National University Medical School, 634-18, Keumam-Dong, Jeonju-Shi, Chonbuk Republic of Korea; 3 Department of Orthopaedic Surgery and Biochemistry, Research Institute of Clinical Medicine of Jeonbuk National University-Biomedical Research Institute of Jeonbuk National University Hospital and Research Institute for Endocrine Sciences, Jeonbuk National University Medical School, Jeonju, Republic of Korea

**Keywords:** Molecular medicine, Musculoskeletal system, Medical research, Inflammation, Trauma

## Abstract

Posttraumatic osteoarthritis is primarily characterized by articular cartilage destruction secondary to trauma or fracture events. Even while intra-articular scar tissue can be observed following ankle fractures, little is known about its nature and molecular events linking its biological activity and cartilage deterioration. Here, we investigated scar tissue's histological and molecular characteristics, and its relationship with localized articular cartilage alterations consistent with early osteoarthritic degeneration. Intra-articular scar tissues from sixty-two patients who underwent open reduction internal fixation for ankle fracture were obtained at hardware removal time (6–44 months after fracture). Histological analysis demonstrated that scar tissue has the nature of fibrosis with fibrous tissue hyperplasia, fibroblast proliferation, and chondrometaplasia. These fibrous scar tissues showed overexpressed pro-inflammatory cytokines and high mRNA expression levels of osteoarthritis-related markers (cytokines, chemokines, and enzymes) compared to the normal synovium. Furthermore, those transcriptional levels were significantly correlated with the grade of talar chondral degeneration. Our findings suggest that following an ankle fracture, the intra-articular fibrous scar tissue exhibits high catabolic and inflammatory activity, which has a long-lasting negative impact correlated to cartilage deterioration in the development of posttraumatic osteoarthritis.

## Introduction

Characterized by a progressive loss of standard structure and function of the articular cartilage, osteoarthritis (OA) is the most typical deteriorative joint disorder that can cause joint pain and disability, which can lead to complete anatomic and functional joint destruction^1^. Unlike the knee and hip, degenerative OA rarely influences the ankle joint^2^. Nevertheless, multiple clinical and epidemiologic studies have shown that previous trauma is the most prevalent cause of ankle OA^3^. Posttraumatic osteoarthritis (PTOA) accounts for about 80–90% of ankle osteoarthritis^4^. Researchers have investigated the etiology and pathomechanic basis of PTOA and concluded that ankle fracture is the most common predisposing factor^5^. Several risk factors, such as the type and severity of the fracture, trauma-related cartilage damage extent, fracture reduction, and joint congruence, for the occurrence of ankle PTOA have been pointed out^6^. Even though fractures can be anatomically reduced by surgery, there is still a possibility for PTOA development. Inadequate knowledge exists regarding the risk factors and pathomechanisms that contribute to the degeneration of the ankle following an injury^1^.

An intra-articular fibrous scar tissue is established in 73% of all cases after surgical reduction of ankle fracture^7^. It may form adhesions within joint capsules, the contracture of tendons and bursa around the joint, causing reduced range of motion and pain^8^. The hypertrophic formation of this tissue can induce arthrofibrosis and cause impingement syndromes of the ankle that may lead to articular dysfunction^9–12^. Nevertheless, little is known about the molecular events linking the metabolic activity of fibrous scar tissue and articular cartilage degradation. The previous studies demonstrated that the expression pattern of multiple pro-inflammatory cytokines, chemokines, and matrix-degrading enzymes is an essential consideration when performing molecular characterization of OA^13–15^. Cytokines (IL1β, TNFα, and IL6) can stimulate cartilage matrix degradation by interfering with collagen type 2 and aggrecan synthesis and inducing the production of enzymes such as metalloproteinases (MMPs) and aggrecanases (particularly ADAMTS4 and ADAMTS5) known to have a destructive effect on cartilage components^16–20^. Additionally, chemoattractive cytokines (chemokines) contribute to inflammatory arthritis by recruiting leukocytes and activating granulocytes and mononuclear cells in the synovial membrane and fluid^21^. These molecules are widely known as markers of joint destruction. The catabolic and inflammatory activity of these osteoarthritis-related genes might result in irreversible cartilage damage, which vitally partakes in the development and progression of PTOA^22^. Following an ankle fracture, the intra-articular fibrous scar tissue was formed within several months and may persist longer^7^. Prior studies demonstrated that the fibrous scar tissue consisted of synovial tissue with extensive fibrosis, which could potentially lead to unfavorable surgical outcomes^7–9^. However, no reports have described the relationship between the metabolic activity of intra-articular scar tissue and the development of ankle PTOA.

This study aims to investigate the histological characteristics and inflammatory activity of intra-articular scar tissue formed after ankle fractures. The second goal was to quantify the mRNA expression of genes for inflammatory cytokines and chemokines, degradative enzymes in this scar tissue. These genes are potentially important in developing secondary osteoarthritis. The other purpose was to analyze the relative expression of these target genes in scar tissue harvested at various stages of cartilage deterioration of the ankle joint. We hypothesized that this scar tissue has the nature of fibrosis and would express catabolic and inflammatory activity in correlation with the stage of cartilage degeneration in the progression of posttraumatic osteoarthritis.

## Results

### Patient demographics

This study includes 62 ankle fracture patients whose intra-articular fibrous scar tissue was observed and collected through second-look arthroscopy at the time of hardware removal surgery, which was at a mean of 14.2 months (range, 6 to 44 months) after the index surgery for ankle fracture (Supplementary Table S1). Of these 62 patients, 23 were women and 39 were men. Their mean age was 50.3 ± 16.2 years (range, 18 to 80 years). Their mean BMI was 26.3 ± 4.0 (range, 18.6 to 39.9).

The etiologic analysis showed lateral malleolar fractures were the most common in this research (30.6%, 19 cases), followed by trimalleolar fractures accounting for 22.6%. The bimalleolar and Pilon-type fractures, each accounted for 16.1% (Supplementary Table S2).

### The intra-articular scar tissue is identified through a second-look arthroscopy

**Figure 1:**
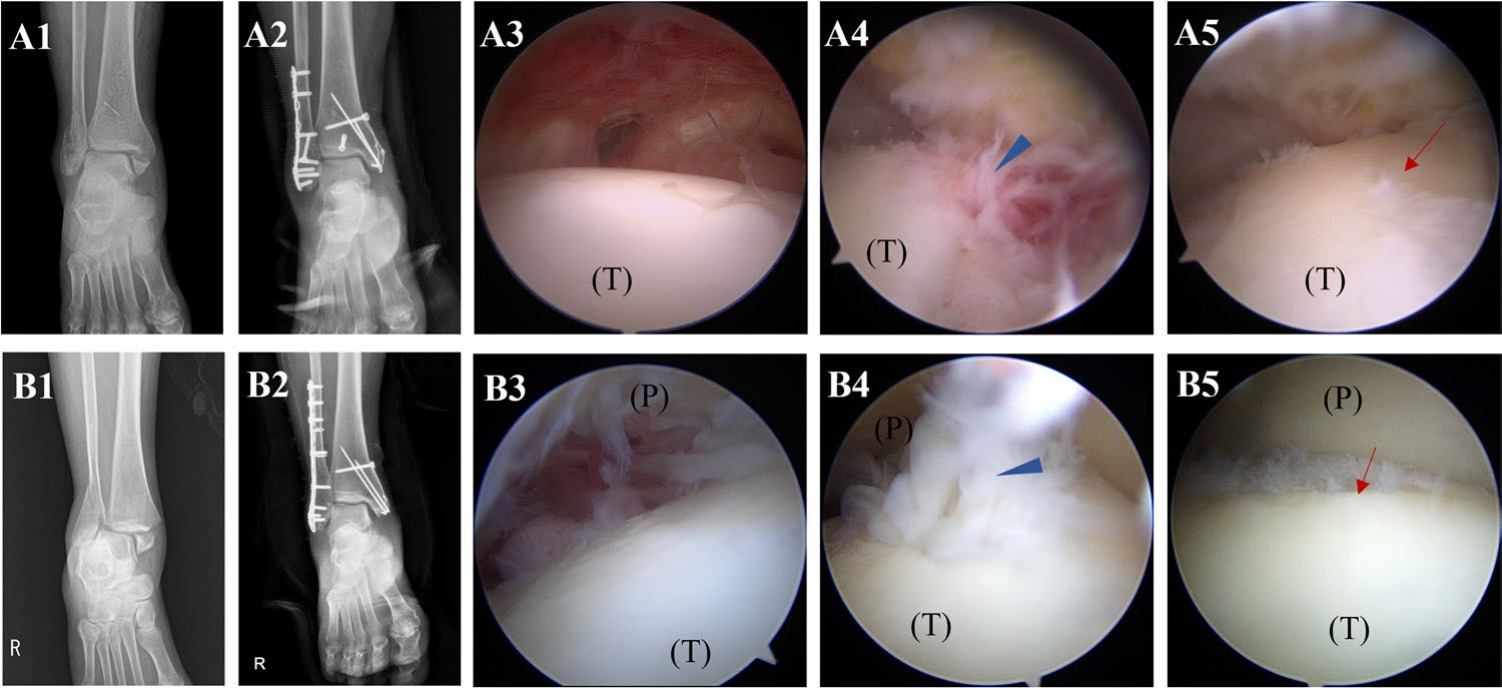
The second-look arthroscopic pictures show the growth of intra-articular scar tissue following trimalleolar fracture (**A1**–**A5**) and bimalleolar fracture (**B1**–**B5**). (**A1**, **B1**) Preoperative X-ray. (**A2**, **B2**) Postoperative X-ray shows good reduction and internal fixation. (**A3**, **B3**) The initial intact cartilage at fractured time**.** (**A4**, **B4**) The second-look arthroscopic finding depicts scar tissue (arrowhead) formed extensively and adhered to the talar dome. (**A5**, **B5**) The underlying degenerative cartilage damage (arrow) is exposed after removing scar tissue. (P): Tibial plafond, (T): Talus.

All of the fractured ankles had intra-articular scar tissue defined by second-look arthroscopy. This scar tissue was extensively produced, cohered with the surrounding structure, partially covering the ankle joint's cartilage surface: talar dome, tibial plafond, and articular aspect of the malleolus. The underlying deteriorating cartilage was visible when the scar tissue was removed. The cartilage surface was fibrillated compared to typical surrounding cartilage or original undamaged cartilage (Fig. 1). In this process, undamaged cartilage gets converted to fibrillated cartilage, similar to degenerative osteoarthritis, in a brief period after osteosynthesis for ankle fracture. As a result, the histological and biological activities of scar tissue must be explained concerning the progression of osteoarthritis.

The underlying degenerative cartilage of the talus was clearly seen after arthroscopic debridement. Talar chondral damage was evaluated using the modified Outerbridge classification. There were twenty-three (37.1%) grade-I patients with softening of the articular surface. Nineteen (30.6%) patients had partial-thickness defects and less than half of the whole thickness. They were classified as grade-II. Fourteen (22.6%) patients were classified as grade-III, with partial-thickness defects more than half of the entire thickness. Six (9.7%) patients had full-thickness defects with exposure of the subchondral bone graded level IV (Supplementary Fig. S1).

**Figure 2:**
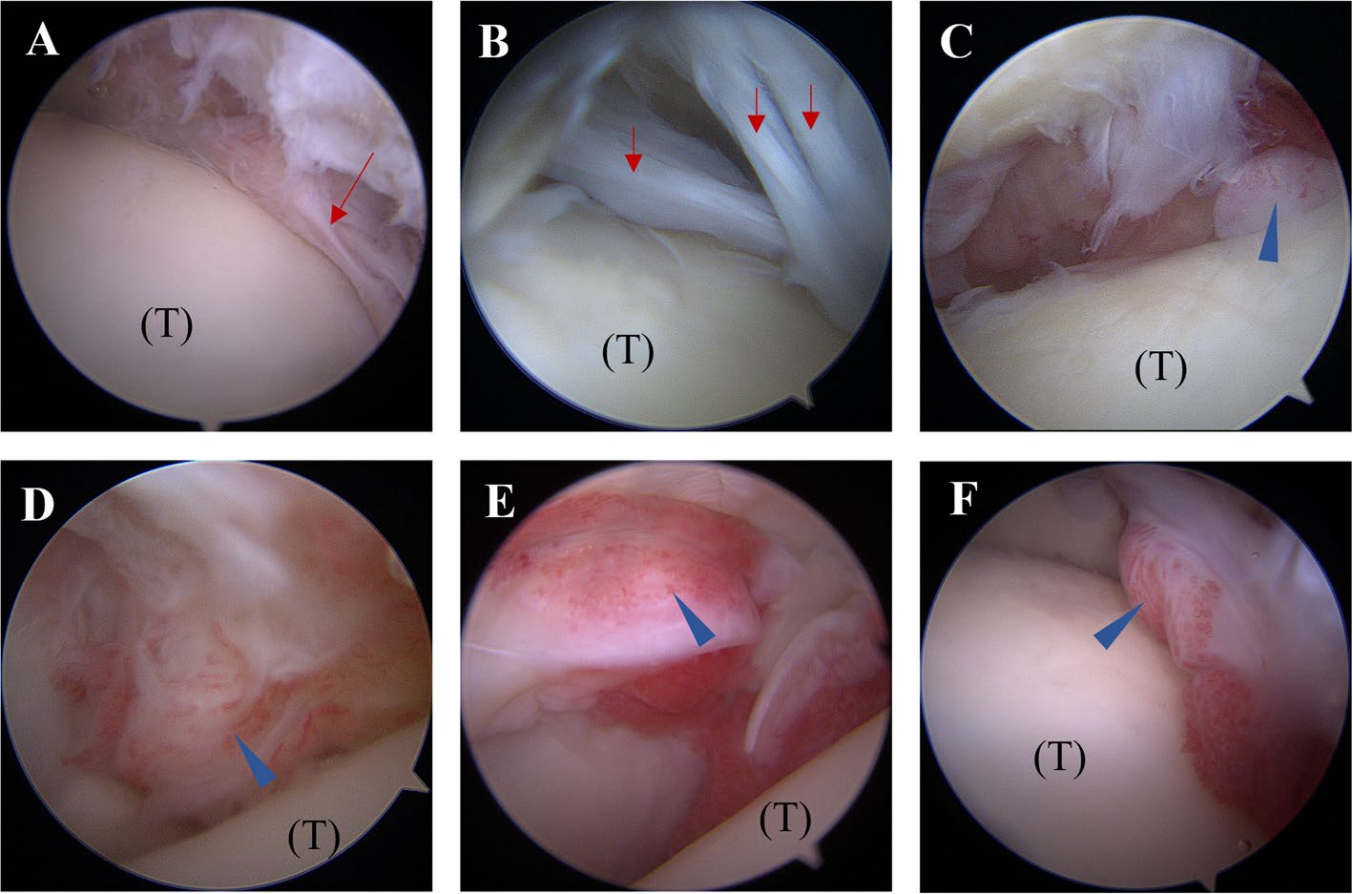
The arthroscopic images demonstrate arthrofibrosis and synovitis formation following ankle fracture. (**A**, **B**) Arthrofibrosis (red arrow). (**C**, **D**) Mild-moderate synovitis. (**E**, **F**) Severe and diffuse synovitis. Blue arrowhead: synovitis, (T): talus.

Arthroscopic findings revealed arthrofibrosis in 15 ankles (24.2%). Macroscopic assessment of synovitis during arthroscopy showed the absence of synovitis in 12 ankles (19.4%), mild to moderate synovitis in 32 ankles (51.6%), and diffuse, severe synovitis in 18 ankles (29.0%) (Fig. 2).

### The intra-articular scar tissue demonstrates fibrosis with chondrometaplasia and overexpresses pro-inflammatory cytokines

**Figure 3:**
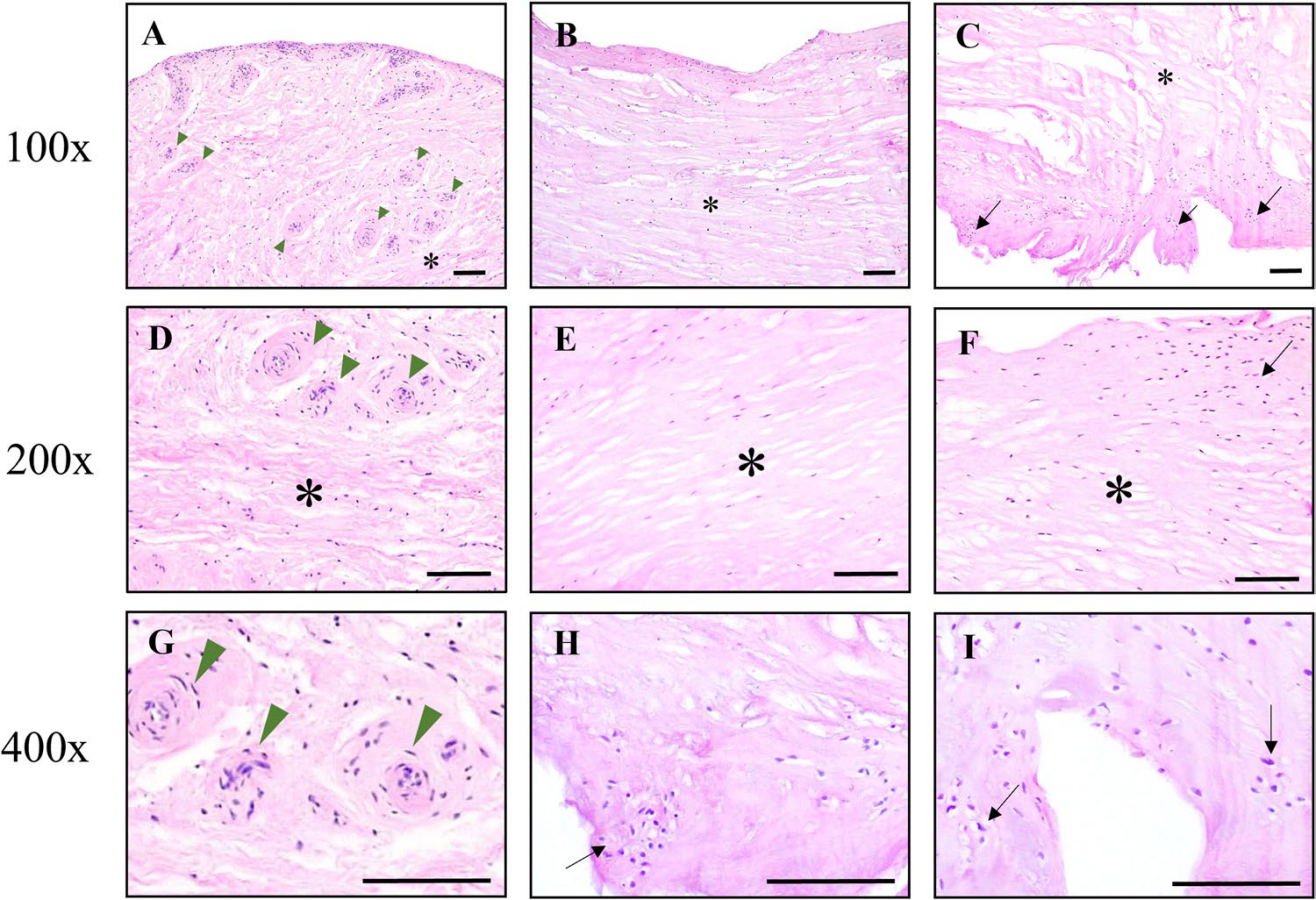
Hematoxylin and Eosin staining of intra-articular scar tissues demonstrated fibrous tissue hyperplasia, clarified by the fibrosis (asterisk), vascular proliferation (arrowhead), and chondrometaplasia (transformation of synovial tissue into hyaline-appearing cartilage). These areas of chondrocytes (black arrow) simultaneously appeared with fibrous tissue (asterisk), showing the chondrometaplasia evident in these intra-articular scar tissue specimens. Scale bars represent 100 μm. Low to high magnification by optical microscopy: (**A**–**C**) 100x, (**D**–**F**) 200x, (**G**–**I**) 400x.

To identify the properties of the scar tissue observed through arthroscopy, we checked the pathological findings after removing the scar tissue using an arthroscopy. Biopsies from the intra-articular scar tissue of fractured ankles demonstrate fibrous tissue hyperplasia with chondrometaplasia. Histopathologically, the removed scar tissue showed excessive fibroblast proliferation and collagen synthesis in the fibrous, which resembles arthrofibrosis characteristics. They showed fibrosis, vascular proliferation, and the transformative form of synovial tissue into hyaline-appearing cartilage (chondrometaplasia) (Fig. 3).

**Figure 4:**
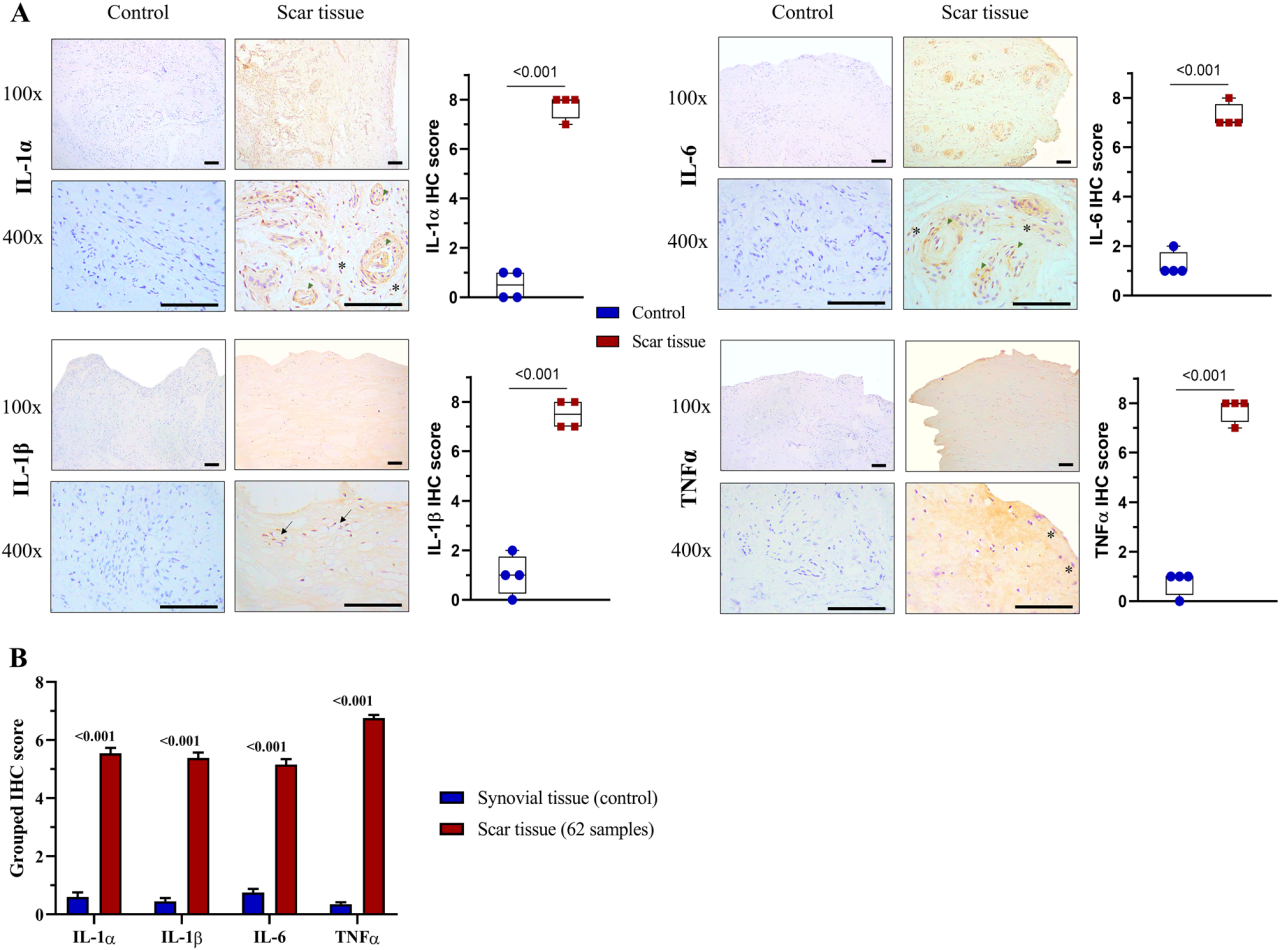
The expression of inflammatory cytokines in intra-articular fibrous scar tissue. Representative immunohistochemical staining for pro-inflammatory cytokines (IL-1α, IL1-β, IL6, TNFα) was conducted in both scar tissue and synovial tissue (control group). (**A**) Positive cells are depicted with cytoplasmic brown staining, with nuclei counterstained using hematoxylin. The high-magnification view highlights positive staining in various cell types, including fibroblasts (asterisk), proliferative vascular endothelial cells (arrowhead), and chondrocytes (arrow). Scale bars indicate 100 μm. The quantification of immunohistochemical staining involves calculating the IHC score for each sample based on the average of four randomly selected fields at 400 × magnification, both for the scar tissue and control samples. The corresponding *p*-values are shown above the horizontal bars. (**B**) The IHC scores for scar tissue (62 samples) are presented, and the associated *p*-values are displayed above each pair of bars. All data were analyzed using the Mann–Whitney U test.

Pro-inflammatory cytokines are the main pathogenic agents influencing cartilage destruction in osteoarthritis^23^. We observed aggressive pro-inflammatory cytokine activities in all scar tissue samples compared with normal synovial tissue. IHC staining revealed the overexpression of IL-1α, IL1-β, IL6, and TNFα in intra-articular fibrous scar tissue (Fig. 4).

### Osteoarthritis-related genes are increased in the intra-articular fibrous scar tissue compared to synovium

**Figure 5:**
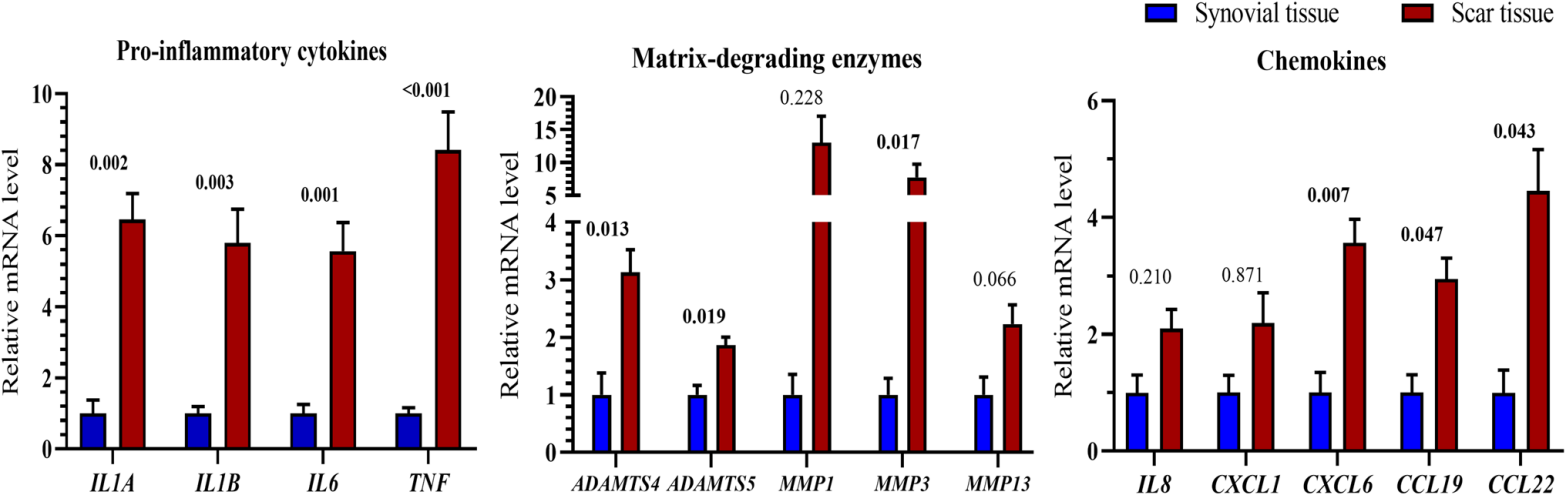
Messenger RNA gene expression levels of pro-inflammatory cytokines (*IL1A, IL1B, IL6,* and *TNF*), matrix-degrading enzymes (*ADAMTS4, ADAMTS5, MMP1, MMP3,* and *MMP13*), and chemokines (*IL8, CXCL1, CXCL6, CCL19,* and *CCL22*) in intra-articular scar tissues procured from 62 ankles underwent fracture compared with those in normal synovial tissues. Expression levels were determined using quantitative real-time polymerase chain reaction with GAPDH (glyceraldehyde 3-phosphate dehydrogenase) as the reference. The *p-*values are indicated above each bar pair. Data were analyzed by the Mann–Whitney U test.

Because synovial tissue inflammation has a role in the pathophysiology of OA, we compared OA-related gene expressions between scar tissue and synovium^24^. The expression levels of all candidate pro-inflammatory cytokines, matrix-degrading enzymes, and chemokines were found to be higher in the fibrous scar tissue of fractured ankles (from 62 patients) when compared to the synovial samples (from 10 control subjects) (Fig. 5). There were remarkable increments in the expression of pro-inflammatory cytokine genes (*p* < 0.01): interleukin-1α (*IL-1A*) (6.5-fold), interleukin-1β (*IL-1B*) (5.8-fold), interleukin-6 (*IL6*) (5.6-fold), and tumor necrosis factor-alpha (*TNF*) (8.4-fold). These differences also reached significance (*p* < 0.05) for the chemokine gene: *CXCL6, CCL19,* and *CCL22*. In addition, expression levels of matrix metalloproteinase enzyme *MMP3* and aggrecanase enzymes (*ADAMTS4, ADAMTS5*) known as indicators that could promote cartilage degrading activity, were also significantly higher in fractured ankles than in synovium (*p* < 0.05).

### Gene expression levels of osteoarthritis-related markers in fibrous scar tissues are markedly correlated with the severity of cartilage degeneration

**Figure 6:**
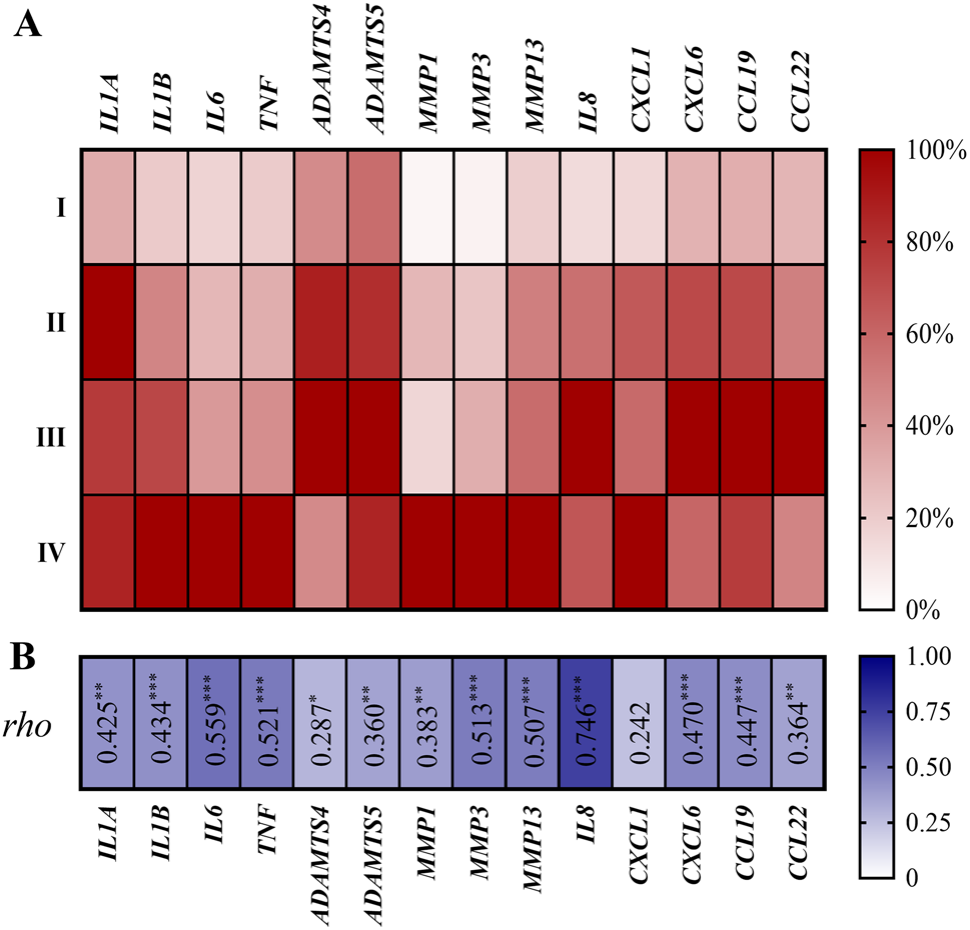
Heat map visualization of the mRNA expression of osteoarthritis-related markers in correlation with the grade of cartilage degradation. (**A**) Relative mRNA levels of the 14 target genes change response to Outerbridge classification of cartilage degeneration. The genes are represented in the columns, the Outerbridge grade (I-IV) is represented in the rows, and the gene expression levels are normalized in percentage (the largest value of relative mRNA expression level in each target gene is defined as 100%). (**B**) The correlation between relative mRNA level in intra-articular fibrous scar tissue versus chondral damage grade using Spearman's rank correlation coefficient. *Rho*-value is indicated inside the cells (*p < 0.05, **p < 0.01, ***p < 0.001)*.*

There was a highly significant correlation between the grade of cartilage degradation and relative mRNA levels of all target genes, except *CXCL1* (Fig. 6, Supplementary Table S3). The more severe the chondral damage was, the higher the relative mRNA level. To clarify the relationship between the articular cartilage breakdown and the gene expression of osteoarthritis-related genes, we reorganized 62 cases into two groups depending on the severity of talar chondral damage. The no chondrosis (mild) group showed no evidence concerning chondrosis, meaning Outerbridge grade I (n = 23). Meanwhile, the chondrosis (more severe) group contained cases with an arthroscopic finding of at least grade II articular cartilage damage in the ankle (n = 39). The results revealed that gene expression levels of all pro-inflammatory cytokines, all matrix-degrading enzymes, and chemokines (except *CXCL1*) were significantly (*p* < 0.05) higher in the "chondrosis" grade than in the "no chondrosis" grade (Supplementary Fig. S2). However, there was no significant relationship between the OA-related markers of scar tissue with the time difference in scar tissue obtained after trauma (except *TNF)*, the fracture pattern (except *IL1A, IL1B, MMP13*, and *IL8*), and the degree of synovitis observed on arthroscopy (Supplementary Table S4, S5, Supplementary Fig. S3).

## Discussion

The formation of intra-articular scar tissue was defined through the second-look arthroscopy at several months (from 6 to 44) following ankle fracture. The H&E staining provided a comprehensive picture of the microanatomy of the scar tissue, which is composed of fibrous tissue developing from synovial hypertrophy. Histological analysis of this fibrous scar tissue illustrated fibrosis, vascular proliferation, and chondrometaplasia, which are arthrofibrosis characteristics^25^. The pro-inflammatory cytokines overexpression was indicated in this scar tissue. Our study shows an overall association between osteoarthritis-related gene expression in scar tissue and the presence of early chondrosis in the ankle articular cartilage as measured by arthroscopy. Furthermore, cartilage degeneration was observed underneath the scar tissue, suggesting that this fibrous scar tissue structure could obliterate the joint space by becoming interposed and induce cartilage damage while in ankle motion. The most important finding of our study is the corroboration of an earlier discovery of molecular biological effect of intra-articular fibrous scar tissue in ankles. These findings support a continuous detrimental effect on secondary cartilage degeneration in posttraumatic osteoarthritis progression.

Several reasons supporting the possibility that the fibrous scar tissue of the ankle joint after fracture might be involved in the worsening of osteoarthritis can be found in the properties of scar tissue and other similar joints. First, fibrous scar tissue is a form of fibrosis^26^. Typical pathogenic pathways transpire in fibrosis of organs or tissues^25^. Immunology and rheumatology research studies have demonstrated that dysfunction of wound healing processes can lead to fibrosis with a significant association with inflammatory cytokines, chemokines, and enzymes^27,28^. Notably, Remst et al.^26^ have reported that over half of patients with OA have synovial fibrosis. Other studies have also discovered an association between OA and fibrosis^8^. Furthermore, many inflammatory cytokines such as TNFα, IL1, IL6, and matrix-degrading enzymes (MMPs) have been proven to be related to the progression of fibrosis^8,29^. These cytokines' overexpression can lead to fibroblast activation, increased collagen type VI synthesis, and prolongation of inflammatory response, resulting in fibrous tissue hyperplasia^9^. Aggrecanases and metalloproteinase are major matrix-degrading enzymes implicated in articular cartilage degradation^13,19,30^. By possible interactions with other inflammatory factors expressed within the osteoarthritic joint, chemokines are responsible for inflammation, leukocyte infiltration, catabolic cascades, and cartilage destruction^14^.

Second, histologic imaging revealed that scar tissue is a transformative form of synovial tissue with fibrosis, vascular proliferation, and chondrometaplasia. Thus, the relationship between fibrous scar tissue and osteoarthritis can be considered through the case of synovial tissues. Our results are consistent with previous research in which arthroscopy-based limited evidence for a connection between early degenerative alterations in the articular cartilage and gene expression in the synovium was discovered. In hip joints, elevation expression of pro-inflammatory cytokine TNF in the synovium might predict OA progression after hip arthroscopy^31^. Abe et al.^30^ have discovered that expression levels of TNFα, IL1β, and IL6 are significantly higher in hip joint fluids of patients with severe OA than in those of patients with early OA. A recent study on the pattern of inflammation in the cartilage and the synovium of patients with femoroacetabular impingement (FAI) undergoing hip arthroscopy has shown that expression levels of inflammation-related proteins are increased in hip OA secondary to FAI with inflammatory evidence observed in the synovium^32^. Hence, elevated gene expression levels of these cytokines, enzymes, and chemokines in intra-articular fibrous scar tissues suggest that their metabolic activity might play a key role in subsequent ankle joint osteoarthritis. It has also been reported that arthroscopic fibrous scar tissue debridement may reduce posttraumatic osteoarthritis development, consistent with our findings^33,34^.

Arthroscopic surgery is vital for confirming the macroscopic assessment of synovitis^35,36^. Mounting evidence suggests that synovitis and the resultant pro-inflammatory mediators are essential in the pathogenesis of OA with effects on articular cartilage^37^. At second-look arthroscopy, findings of synovitis were confirmed in 80.6%, consistent with injury and the development of talar chondrosis (62.9%) that also contributes to OA after ankle fracture. However, no relationship between scar tissue's gene expression and arthroscopic findings of synovitis was found. This result suggests that the catabolic activity of scar tissue and synovial inflammation have independent effects on degenerative changes in a fractured ankle. It nevertheless did not exclude the intact synovium's potential contribution to the cartilage degeneration in ankle PTOA. In order to clarify the role of individual inflammation source, further research is required to determine the OA-related gene expression characteristics of both intra-articular scar tissue and the intact synovium. Another important aspect of this study is no correlation between the osteoarthritis-related gene expression in scar tissue and time after ankle fracture. This event demonstrates that intra-articular scar tissue possesses time-independent catabolic and inflammatory activities. As a result, these persistent detrimental behaviors can have a long-term impact on the course of traumatic arthritis, both before and after the bone union. Meanwhile, previous studies have shown that the initial inflammatory response following intra-articular fracture might induce synovial catabolism and cartilage degradation^38–41^.

In this study, we evaluated the osteoarthritis status of all patients through second-look arthroscopy during hardware removal surgery. Arthroscopic evaluation is considered the benchmark to assess the cartilage injury and macroscopic finding of synovitis since it allows direct visualization of the lesion, giving a way to perform early diagnosis of PTOA, which generally precede radiographic osteoarthritic changes^42^. These discoveries provide the foundation that distinct cytokines, enzymes, or chemokines produced through fibrous scar tissue could be used as candidate biomarkers for diagnosing and staging posttraumatic pre-osteoarthritic or early osteoarthritic disorders.

This study has certain limitations. First, this study only included a small number of patients, and there were discrepancies in the sample sizes for each group of chondral damage. Second, although we found that fibrous scar tissue gene expression in the fractured ankle might facilitate the osteoarthritis cascade, we did not determine each factor's specific molecular or mechanistic role in the pathophysiology of PTOA. Future investigations should concentrate on the mechanisms of the osteoarthritis process induced by scar tissue. Third, for comparison, control synovial tissues were from abnormal ankles (ligament injury), not healthy, uninjured ankles. Fourth, we did not measure the levels of inflammatory cytokines, matrix-degrading enzymes, and chemokines in the ankle synovial fluid of patients, which could potentially relate to gene expression in intra-articular scar tissue. Finally, we did not always do the initial arthroscopy directly after the fracture. Thus, we are unable to evaluate all cartilage damage immediately following trauma. This study represents the first step in molecular characterizing the posttraumatic intra-articular fibrous scar tissue and analyzing the relationship with the cartilage degeneration in the ankle joint. So, the clinical outcome measurements were beyond the scope of this short-term follow-up study. Future investigations involving more cases with long-term evaluations are required to fully understand how the intra-articular fibrous scar tissue might contribute to the progression and development of PTOA. Despite these limitations, this study represents the first to analyze scar tissue formation osteoarthritis-related gene expression following ankle fracture. Further investigation of gene expression in scar tissues may reveal innovative disease markers for early diagnosis of PTOA and the potential discovery of new therapeutic targets to delay or prevent osteoarthritis after ankle fracture.

In conclusion, this study demonstrates that the intra-articular scar tissue formation following ankle fracture has the nature of fibrosis with fibrous tissue hyperplasia, fibroblast proliferation, and chondrometaplasia. This fibrous scar tissue exhibits high catabolic and inflammatory activity, which has a long-lasting negative impact correlated to cartilage deterioration of the ankle. These findings prove a continuous detrimental effect on secondary cartilage degeneration in posttraumatic osteoarthritis progression.

## Methods

### Patients and tissue samples

All patients who underwent open reduction and internal fixation (ORIF) surgery for ankle fractures at our hospital were asked to consider a second-look arthroscopic surgery during the hardware removal surgery. Even when hardware-related pain or unclear ankle discomfort was modest, these treatments were typically only performed after a non-eventful course and radiographically confirmed bone union, about one year after the fracture. From March 2020 to August 2022, the intra-articular scar tissues from sixty-two ankles (62 patients) were observed and collected intraoperatively with arthroscopy instruments. The arthroscopic procedure was performed with a 2.7-mm, 30° oblique arthroscope using standard anterolateral and anteromedial portals. All surgical procedures were conducted by a single senior foot and ankle surgeon (KBL). Exclusion criteria were those with primary or secondary infections and systemic inflammatory diseases such as rheumatoid arthritis, anti-inflammatory medication, or injuries connected with a Charcot's foot. Patients were also excluded if there was any sign of radiographic osteoarthritis finding at the fractured time.

The Institutional Review Board (IRB) of Jeonbuk National University Research Council approved all processes in this study (Approval number: CUH 2021-10-032). All patients involved provided informed consent for this study.

### Arthroscopic evaluation

The second-look ankle arthroscopic visualization was performed to evaluate the intra-articular scar tissue formation, macroscopic assessment of synovitis, and the chondral degeneration at hardware removal time. Articular cartilage damage of the talus was assessed and graded twice using the modified Outerbridge classification^43,44^ (Supplementary Table S6) by one of the authors (KBL) and an independent foot and ankle fellow observer. The ankle was allotted a grade based on the most severe chondral damaged area.

### Fracture classification analysis

According to the severity of the fractures, patients have divided into three groups: stage 1 patients had an isolated fibula or medial malleolus fracture, stage 2 patients had any bimalleolar fractures (fibula plus medial malleolus fractures), and trimalleolar fractures or any fracture including the posterior malleolus was classified as stage 3^45^.

### Histology

All labeled specimens in sterile phosphate-buffered saline solution in screw-cap containers were sent to the hospital's biobank from the operating room. In the laboratory, these tissues were weighed and washed with Dulbecco's Phosphate-Buffered Saline (DPBS) solution. Biopsies from the scar tissues were immediately placed in formalin, paraffin-embedded, sectioned, and stained using hematoxylin and eosin (H&E). Immunohistochemistry (IHC) staining used the HRP/DAB (ABC) detection IHC kit with specific primary antibodies anti-IL-1α (ab9614), anti-IL-1β (CS-12242), anti-IL6 (NB600-1131), or anti-TNFα (sc-52746). Immunostained slides were scored the same as previously described^46,47^. First, a proportion score was assigned from 0 to 5, representing the expected proportion of positive-staining cells (0, none; 1, < 1/100; 2, 1/100 to 1/10; 3, 1/10 to 1/3; 4, 1/3 to 2/3; and 5, > 2/3. Next, an intensity score was allocated from 0 to 3, representing the average intensity of positive cells (0, no expression; 1, weak expression, 2, moderate expression; and 3, strong expression). The proportion and intensity scores were combined to obtain a total immunohistochemical staining score (IHC score) ranging from 0 to 8. The IHC score of each sample was defined by the average of four randomly chosen fields at 400 × magnification. Images were acquired using a Leica DM750 microscope (Leica, Wetzlar, Germany). The rest of the specimens were put in 2-mL tubes, centrifuged, dehydrated, and stored at -80ºC until used for total RNA extraction.

### Quantitative real-time PCR with reverse-transcription analysis

All frozen specimens were thawed and homogenized directly in TRIzol reagent (Invitrogen). According to the manufacturer's instructions, total RNA was extracted, precipitated with isopropanol, and dissolved in RNase-free water (Qiagen, Valencia, California). Next, total RNA was reverse-transcribed into cDNA using random hexamer primers and the First-Strand Synthesis System (Invitrogen) as stated in the instructions provided by the manufacturer. Quantitative Real-Time Polymerase Chain Reaction (qPCR) was carried out in 384-well plates using an ABI Prism 7900HT Sequence Detection System (Applied Biosystems, Foster City, CA, USA). RNAs from individual specimens were analyzed separately. For comparison, ten synovial tissue samples (control samples) were also obtained from patients who had undergone arthroscopy surgeries to remedy symptomatic anterior talofibular ligament (ATFL) injury without a history of ankle fracture, cartilage injury, or osteoarthritis (Supplementary Table S7, S8). The relative fold gene expression level of each sample was calculated by using the formula *X* = 2^−∆∆*Ct*^, where ∆∆Ct = ∆Ct (experimental sample) – ∆Ct (Control average) and ∆Ct = Ct (target gene) – Ct (housekeeping gene). ∆Ct Control average was calculated by arithmetic average ∆Ct of the 10 control samples.

Genes selected for evaluation included genes encoding pro-inflammatory cytokines, matrix degradation enzymes, and chemokines known to play a vital role in osteoarthritis or joint degradation. Gene expression was normalized to the level of glyceraldehyde 3-phosphate dehydrogenase (GAPDH) as a housekeeping gene. Primer sequences are listed in Supplementary Table S9.

### Statistical analysis

All statistical analyses were conducted using SPSS software version 26.0 (IBM, Armonk, NY, USA). Descriptive statistics, including arithmetic means, standard error of the mean, and ranges, were calculated using standard formulas. The Kolmogorov–Smirnov test was utilized to ascertain if the data were normally distributed. Comparisons between the two groups were gauged using an independent t-test (Student's t-test) in normally distributed continuous variables. For non-normally distributed data, the Mann–Whitney U test was used to analyze differences. The chi-square or Fisher exact test was used for dichotomous variables to analyze differences. We used a Kruskal–Wallis test to compare more than two groups. Correlation analyses were executed using Spearman's rank correlation test. Differences with a *p*-value < 0.05 were considered statistically significant. A statistician reviewed all aspects of statistical analyses.

### Ethical approval

The study was conducted following the Declaration of Helsinki and approved by the Institutional Review Board (IRB) of Jeonbuk National University Research Council (Approval number: CUH 2021-10-032). All patients involved provided informed consent for this study.

### Supplementary Information

Supplementary Tables.

Supplementary Figures.

## Data Availability

The datasets used and analyzed during this study are available from the corresponding author on reasonable request.
