# Supplementary material for: Continuous detrimental activity of intra-articular fibrous scar tissue in correlation with posttraumatic ankle osteoarthritis
Source: Sci Rep. 2023 Nov 16;13:20058. doi: 10.1038/s41598-023-47498-7 (PMC10654697; doi:10.1038/s41598-023-47498-7)
Supplement: Supplementary file 1 — Supplementary Tables. [file 41598_2023_47498_MOESM1_ESM.docx]

**Supplementary Table S1.** Patient demographic and clinical data of ankle fracture group

| **No.** | **Sex** | **Age**  **(year)** | **BMI (kg/m^2^)** | **2^nd^ look Outerbridge grade** | **2^nd^ look**  **Synovitis** | **Type of Fracture** | **Fracture stage** | **Time after fracture (month)** |
| --- | --- | --- | --- | --- | --- | --- | --- | --- |
| 1 | M | 39 | 26.7 | I | S | Trimalleolar | 3 | 12 |
| 2 | M | 55 | 28.1 | I | M | Medial Malleolus | 1 | 10 |
| 3 | M | 67 | 23.9 | I | S | Trimalleolar | 3 | 8 |
| 4 | F | 66 | 26.0 | I | M | Fibula | 1 | 8 |
| 5 | M | 37 | 33.6 | I | No | Fibula | 1 | 12 |
| 6 | M | 28 | 27.0 | I | M | Medial Malleolus | 1 | 6 |
| 7 | M | 21 | 18.6 | I | M | Fibula | 1 | 9 |
| 8 | F | 55 | 22.2 | I | M | Bimalleolar | 2 | 28 |
| 9 | M | 18 | 20.0 | I | M | Fibula | 1 | 12 |
| 10 | M | 50 | 27.0 | I | S | Pilon | 3 | 11 |
| 11 | F | 47 | 23.6 | I | M | Fibula | 1 | 7 |
| 12 | M | 80 | 18.6 | I | No | Bimalleolar | 2 | 12 |
| 13 | F | 66 | 24.4 | I | M | Fibula | 1 | 12 |
| 14 | M | 31 | 27.0 | I | No | Fibula | 1 | 12 |
| 15 | F | 62 | 29.4 | I | M | Fibula | 1 | 12 |
| 16 | M | 60 | 27.4 | I | S | Fibula | 1 | 9 |
| 17 | F | 62 | 26.5 | I | No | Bimalleolar | 2 | 12 |
| 18 | M | 62 | 21.5 | I | S | Bimalleolar | 2 | 6 |
| 19 | M | 50 | 28.3 | I | M | Fibula | 1 | 17 |
| 20 | M | 38 | 25.9 | I | M | Pilon | 3 | 24 |
| 21 | M | 31 | 27.0 | I | No | Fibula | 1 | 12 |
| 22 | M | 26 | 27.7 | I | M | Medial Malleolus | 1 | 14 |
| 23 | M | 18 | 28.0 | I | S | Trimalleolar | 3 | 9 |
| 24 | M | 20 | 25.8 | II | No | Posterior malleolus | 3 | 38 |
| 25 | M | 62 | 24.2 | II | M | Medial Malleolus | 1 | 18 |
| 26 | F | 42 | 24.7 | II | M | Bimalleolar | 2 | 44 |
| 27 | M | 22 | 27.7 | II | M | Pilon | 3 | 10 |
| 28 | M | 50 | 26.6 | II | M | Trimalleolar | 3 | 25 |
| 29 | M | 68 | 29.4 | II | M | Pilon | 3 | 11 |
| 30 | M | 31 | 21.6 | II | M | Trimalleolar | 3 | 8 |
| 31 | M | 42 | 27.4 | II | S | Bimalleolar | 2 | 14 |
| 32 | M | 62 | 29.0 | II | No | Trimalleolar | 3 | 10 |
| 33 | F | 35 | 24.0 | II | M | Fibula | 1 | 19 |
| 34 | M | 60 | 26.8 | II | M | Medial Malleolus | 1 | 10 |
| 35 | M | 53 | 23.6 | II | S | Bimalleolar | 2 | 16 |
| 36 | F | 68 | 21.3 | II | M | Fibula | 1 | 11 |
| 37 | F | 63 | 28.2 | II | M | Bimalleolar | 2 | 8 |
| 38 | F | 53 | 39.9 | II | S | Trimalleolar | 3 | 11 |
| 39 | M | 64 | 30.9 | II | S | Fibula | 1 | 8 |
| 40 | F | 69 | 20.6 | II | No | Fibula | 1 | 10 |
| 41 | F | 59 | 26.8 | II | No | Fibula | 1 | 10 |
| 42 | F | 49 | 21.2 | II | M | Trimalleolar | 3 | 10 |
| 43 | M | 64 | 33.4 | III | M | Bimalleolar | 2 | 13 |
| 44 | F | 64 | 22.3 | III | S | Trimalleolar | 3 | 10 |
| 45 | M | 45 | 24.2 | III | M | Trimalleolar | 3 | 12 |
| 46 | F | 63 | 21.1 | III | S | Bimalleolar | 2 | 8 |
| 47 | F | 66 | 28.0 | III | S | Posterior malleolus | 3 | 6 |
| 48 | M | 56 | 32.3 | III | M | Fibula | 1 | 11 |
| 49 | F | 60 | 23.4 | III | M | Pilon | 3 | 12 |
| 50 | F | 60 | 33.1 | III | S | Trimalleolar | 3 | 8 |
| 51 | M | 21 | 27.6 | III | S | Medial Malleolus | 1 | 28 |
| 52 | M | 63 | 27.9 | III | No | Medial Malleolus | 1 | 6 |
| 53 | F | 36 | 32.8 | III | M | Pilon | 3 | 31 |
| 54 | F | 19 | 26.9 | III | S | Pilon | 3 | 12 |
| 55 | F | 67 | 20.4 | III | M | Trimalleolar | 3 | 14 |
| 56 | M | 55 | 28.8 | III | M | Trimalleolar | 3 | 12 |
| 57 | M | 56 | 28.2 | IV | No | Pilon | 3 | 25 |
| 58 | F | 62 | 22.7 | IV | S | Pilon | 3 | 11 |
| 59 | M | 58 | 27.7 | IV | M | Fibula | 1 | 10 |
| 60 | M | 70 | 23.7 | IV | M | Trimalleolar | 3 | 23 |
| 61 | M | 55 | 27.1 | IV | S | Pilon | 3 | 44 |
| 62 | M | 36 | 30.0 | IV | No | Fibula | 1 | 18 |

M: male, F: female. Synovitis: S: severe, M: mild-moderate, No: No synovitis.

**Supplementary Table S2.** Fracture classification.

| **Type of fracture** | **Cases (%)** | **Male/Female** | **Fracture classification** | **Cases (%)** |
| --- | --- | --- | --- | --- |
| Fibula | 19 (30.6) | 11/8 | 1 | 26 (41.9) |
| Medial Malleolus | 7 (11.3) | 7/0 | 1 |  |
| Bimalleolar | 10 (16.1) | 5/5 | 2 | 10 (16.2) |
| Pilon | 10 (16.1) | 6/4 | 3 | 26 (41.9) |
| Trimalleolar | 14 (22.6) | 9/5 | 3 |  |
| Posterior malleolus | 2 (3.3) | 1/1 | 3 |  |
| Total | **62 (100.0)** | **29/23** |  | **62 (100.0)** |

**Supplementary Table S3**. The correlations between relative mRNA level in intra-articular fibrous tissue versus chondral damage grade (Outerbridge classification).

| Genes | Grade I | Grade II | Grade III | Grade IV | Correlation with Chondral Damage | |
| --- | --- | --- | --- | --- | --- | --- |
|  |  |  |  |  | rho | p-value |
| Pro-inflammatory cytokines | | | | | | |
| IL1A | 3.09 ± 0.91 | 9.40 ± 1.55 | 7.26 ± 1.20 | 8.08 ± 1.67 | 0.425 | **0.001** |
| IL1B | 2.47 ± 0.57 | 5.74 ± 1.26 | 8.65 ± 2.30 | 12.02 ± 6.22 | 0.434 | **<0.001** |
| IL6 | 2.81 ± 0.64 | 4.66 ± 1.13 | 6.58 ± 1.02 | 16.60 ± 4.88 | 0.559 | **<0.001** |
| TNF | 4.62 ± 0.50 | 7.22 ± 2.21 | 10.07 ± 1.56 | 22.85 ± 3.96 | 0.521 | **<0.001** |
| Matrix-degrading enzymes | | | | | | |
| ADAMTS4 | 2.01 ± 0.53 | 3.89 ± 0.79 | 4.43 ± 0.79 | 2.02 ± 1.03 | 0.287 | **0.024** |
| ADAMTS5 | 1.38 ± 0.21 | 1.98 ± 0.18 | 2.42 ± 0.38 | 2.07 ± 0.30 | 0.360 | **0.004** |
| MMP1 | 2.11 ± 1.12 | 15.67 ± 8.89 | 8.99 ± 4.73 | 56.09 ± 21.78 | 0.383 | **0.002** |
| MMP3 | 1.49 ± 0.33 | 6.81 ± 3.07 | 9.53 ± 5.73 | 30.17 ± 9.36 | 0.513 | **<0.001** |
| MMP13 | 0.94 ± 0.18 | 2.48 ± 0.34 | 2.85 ± 0.40 | 4.95 ± 2.88 | 0.507 | **<0.001** |
| Chemokines | | | | | | |
| IL8 | 0.57 ± 0.12 | 2.29 ± 0.65 | 4.09 ± 0.87 | 2.71 ± 0.46 | 0.746 | **<0.001** |
| CXCL1 | 0.70 ± 0.12 | 2.93 ± 1.46 | 2.63 ± 0.79 | 4.51 ± 1.73 | 0.242 | 0.058 |
| CXCL6 | 1.75 ± 0.43 | 4.13 ± 0.79 | 5.80 ± 0.84 | 3.47 ± 1.23 | 0.470 | **<0.001** |
| CCL19 | 1.46 ± 0.38 | 3.30 ± 0.73 | 4.65 ± 0.69 | 3.54 ± 1.25 | 0.447 | **<0.001** |
| CCL22 | 2.41 ± 0.77 | 4.16 ± 1.13 | 8.37 ± 1.96 | 4.03 ± 2.31 | 0.364 | **0.004** |

**Supplementary Table S4.** The correlations between relative mRNA level in intra-articular fibrous scar tissue of fractured ankles versus time after fracture (6-44 months).

| Genes | Spearman's rho | p-value |
| --- | --- | --- |
| Pro-inflammatory cytokines | | |
| IL1A | 0.218 | 0.089 |
| IL1B | 0.013 | 0.922 |
| IL6 | 0.108 | 0.403 |
| **TNF** | **0.307** | **0.015** |
| Matrix-degrading enzymes | | |
| ADAMTS4 | 0.103 | 0.428 |
| ADAMTS5 | 0.056 | 0.663 |
| MMP1 | 0.107 | 0.407 |
| MMP3 | 0.040 | 0.757 |
| MMP13 | 0.144 | 0.264 |
| Chemokines | | |
| IL8 | 0.226 | 0.077 |
| CXCL1 | -0.035 | 0.784 |
| CXCL6 | -0.011 | 0.934 |
| CCL19 | 0.088 | 0.497 |
| CCL22 | 0.079 | 0.541 |

**Supplementary Table S5.** The correlations between relative mRNA level in intra-articular fibrous tissue versus fracture type (According to the severity).

| Genes | Stage 1 | Stage 2 | Stage 3 | Correlation with Chondral Damage | |
| --- | --- | --- | --- | --- | --- |
|  |  |  |  | rho | p-value |
| Pro-inflammatory cytokines | | | | | |
| **IL1A** | **3.42 ± 0.69** | **7.03 ± 2.07** | **9.26 ± 1.17** | **0.452** | **<0.001** |
| **IL1B** | **4.00 ± 1.06** | **3.90 ± 1.74** | **8.32 ± 1.82** | **0.296** | **0.019** |
| IL6 | 5.08 ± 1.26 | 4.91 ± 2.14 | 6.29 ± 1.25 | 0.167 | 0.194 |
| TNF | 6.42 ± 1.02 | 9.99 ± 4.03 | 9.79 ± 1.77 | 0.209 | 0.104 |
| Matrix-degrading enzymes | | | | | |
| ADAMTS4 | 2.71 ± 0.63 | 3.39 ± 1.01 | 3.45 ± 0.57 | 0.145 | 0.262 |
| ADAMTS5 | 1.60 ± 0.22 | 2.04 ± 0.35 | 2.06 ± 0.21 | 0.220 | 0.085 |
| MMP1 | 15.63 ± 5.97 | 17.35 ± 15.70 | 8.81 ± 4.64 | 0.139 | 0.282 |
| MMP3 | 5.28 ± 2.53 | 8.06 ± 5.69 | 10.01 ± 3.62 | 0.182 | 0.157 |
| **MMP13** | **1.15 ± 0.17** | **3.37 ± 0.59** | **2.88 ± 0.70** | **0.361** | **0.004** |
| Chemokines | | | | | |
| **IL8** | **1.04 ± 0.20** | **3.15 ± 1.20** | **2.75 ± 0.56** | **0.384** | **0.002** |
| CXCL1 | 1.69 ± 0.43 | 3.65 ± 2.72 | 2.13 ± 0.58 | -0.014 | 0.915 |
| CXCL6 | 3.07 ± 0.56 | 3.55 ± 0.77 | 4.06 ± 0.74 | 0.123 | 0.341 |
| CCL19 | 2.53 ± 0.55 | 3.90 ± 1.21 | 3.00 ± 0.48 | 0.170 | 0.186 |
| CCL22 | 4.13 ± 1.06 | 6.07 ± 2.40 | 4.15 ± 0.99 | 0.157 | 0.222 |

**Supplementary Table S6.** Modified Outerbridge Classification of Articular Cartilage in Arthritis.

| Grade | Description |
| --- | --- |
| 0 | Normal cartilage |
| I | Chondral softening or blistering with intact surface |
| II | Superficial ulceration, fibrillation, or fissuring <50% of depth of cartilage |
| III | Deep ulceration, fibrillation, fissuring, or chondral flap >50% of cartilage without exposed bone |
| IV | Full-thickness wear with exposed subchondral bone |

**Supplementary Table S7.** Patient demographic of control group (anterior talofibular ligament injury without a history of ankle fracture, cartilage injury, or osteoarthritis).

| **Case** | **Sex** | **Age**  **(year)** | **BMI (kg/m^2^)** |
| --- | --- | --- | --- |
| 1 | M | 52 | 28.4 |
| 2 | F | 55 | 26.2 |
| 3 | F | 83 | 29.1 |
| 4 | F | 27 | 24.7 |
| 5 | M | 23 | 37.7 |
| 6 | F | 41 | 33.3 |
| 7 | M | 41 | 23.6 |
| 8 | M | 24 | 26.6 |
| 9 | F | 30 | 23.8 |
| 10 | M | 31 | 27.8 |

**Supplementary Table S8**. Source of samples

|  | **Scar tissue** | **Control** | **P value** |
| --- | --- | --- | --- |
| Ankles (no.) | 62 | 10 |  |
| Sex (male/female) | 39/23 | 5/5 | 0.496 |
| Mean age (range) (yr) | 50.3 (18-80) | 40.7 (23-83) | 0.094 |
| BMI (range) (kg/m^2^) | 26.3 (18.6-39.9) | 23.6 (28.1-37.7) | 0.187 |
| Tissue source | Fibrous tissue | Normal synovium |  |

**Supplementary Table S9**. Essential candidate genes in the potential biological link between fibrous scar tissue and posttraumatic osteoarthritis.

| **NCBI* Accession No.** | **Symbol** | **Gene Name** | **Forward Primer** | **Reverse Primer** | **Metabolic role** |
| --- | --- | --- | --- | --- | --- |
| NM_000575.4 | IL1A | Interleukin 1 alpha | 5′-TGCCTGAGATACCCAAAACC-3′ | 5′-AACAAGTTTGGATGGGCAAC-3′ | Inflammation |
| NM_000576.2 | IL1B | Interleukin 1 beta | 5′-TCCAGGAGAATGACCTGAGC-3′ | 5′-GTGATCGTACAGGTGCATCG-3′ | Inflammation |
| NM_000600.5 | IL6 | Interleukin 6 | 5′-AGAGGCACTGGCAGAAAAC-3’ | 5′-TGCAGGAACTGGATCAGGAC-3′ | Inflammation |
| NM_000594.4 | TNF | Tumor necrosis factor | 5′-AACCTCCTCTCTGCCATCAA-3′ | 5’-ggaagacccctcccagatag-3’ | Inflammation |
| NM_005099.5 | ADAMTS4 | A disintegrin and metalloproteinase with thrombospondin motifs 4 | 5′-GGCTAAAGCGCTACCTGCTA-3′ | 5’-GAGTCACCACCAAGCTGACA-3’ | Degradation (catabolism) |
| NM_007038.5 | ADAMTS5 | A disintegrin and metalloproteinase with thrombospondin motifs 5 | 5′-TACTTGGCCTCTCCCATGAC-3′ | 5′-CTGTGATGGTGGCTGAAGTG-3′ | Degradation (catabolism) |
| NM_002421.4 | MMP1 | Matrix metalloproteinase 1 | 5′-AGGTCTCTGAGGGTCAAGCA-3′ | 5′-CTGGTTGAAAAGCATGAGCA-3′ | Degradation (catabolism) |
| NM_002422.5 | MMP3 | Matrix metalloproteinase 3 | 5′-TGCTTTGTCCTTTGATGCTG-3′ | 5′-GGAAGAGATGGCCAAAATGA-3′ | Degradation (catabolism) |
| NM_002427.3 | MMP13 | Matrix metalloproteinase 13 | 5′-TGGTCCAGGAGATGAAGACC-3′ | 5′-TCCTCGGAGACTGGTAATGG-3′ | Degradation (catabolism) |
| NM_000584.4 | CXCL8  (IL8) | Chemokine (C-X-C motif) ligand 8  (Interleukin 8) | 5′-GAAGGTGCAGTTTTGCCAAG-3′ | 5′-TGTGGTCCACTCTCAATCACTC-3′ | Inflammation |
| NM_001511.4 | CXCL1 | Chemokine (C-X-C motif) ligand 1 | 5′-GGGAATTCACCCCAAGAAC-3′ | 5′-GATGCAGGATTGAGGCAAG-3′ | Inflammation |
| NM_002993.4 | CXCL6 | Chemokine (C-X-C motif) ligand 6 | 5′-GTTTACGCGTTACGCTGAGAG-3′ | 5′-ACTTCCACCTTGGAGCACTG-3′ | Inflammation |
| NM_006274.3 | CCL19 | Chemokine (C-C motif) ligand 19 | 5’-CTGCTGGTTCTCTGGACTTCC-3’ | 5’-AGGGATGGGTTTCTGGGTCA-3’ | Inflammation |
| NM_002990.5 | CCL22  (MDF) | Chemokine (C-C motif) ligand 22  (macrophage-derived chemokine) | 5′-CCCTACGGCGCCAACAT-3′ | 5′-CAGACGGTAACGGACGTAATCA-3′ | Inflammation |
| NM_002046.3 | GAPDH | Glyceraldehyde 3-phosphate dehydrogenase | 5′-ACCCAGAAGACTGTGGATGG-3′ | 5'-GAGGCAGGGATGATGTTCTG-3' | Housekeeping |
| *NCBI = National Center for Biotechnology Information. | | | | | |
