# Supplementary material for: Continuous detrimental activity of intra-articular fibrous scar tissue in correlation with posttraumatic ankle osteoarthritis
Source: Sci Rep. 2023 Nov 16;13:20058. doi: 10.1038/s41598-023-47498-7 (PMC10654697; doi:10.1038/s41598-023-47498-7)
Supplement: Supplementary file 2 — Supplementary Figures. [file 41598_2023_47498_MOESM2_ESM.docx]

**
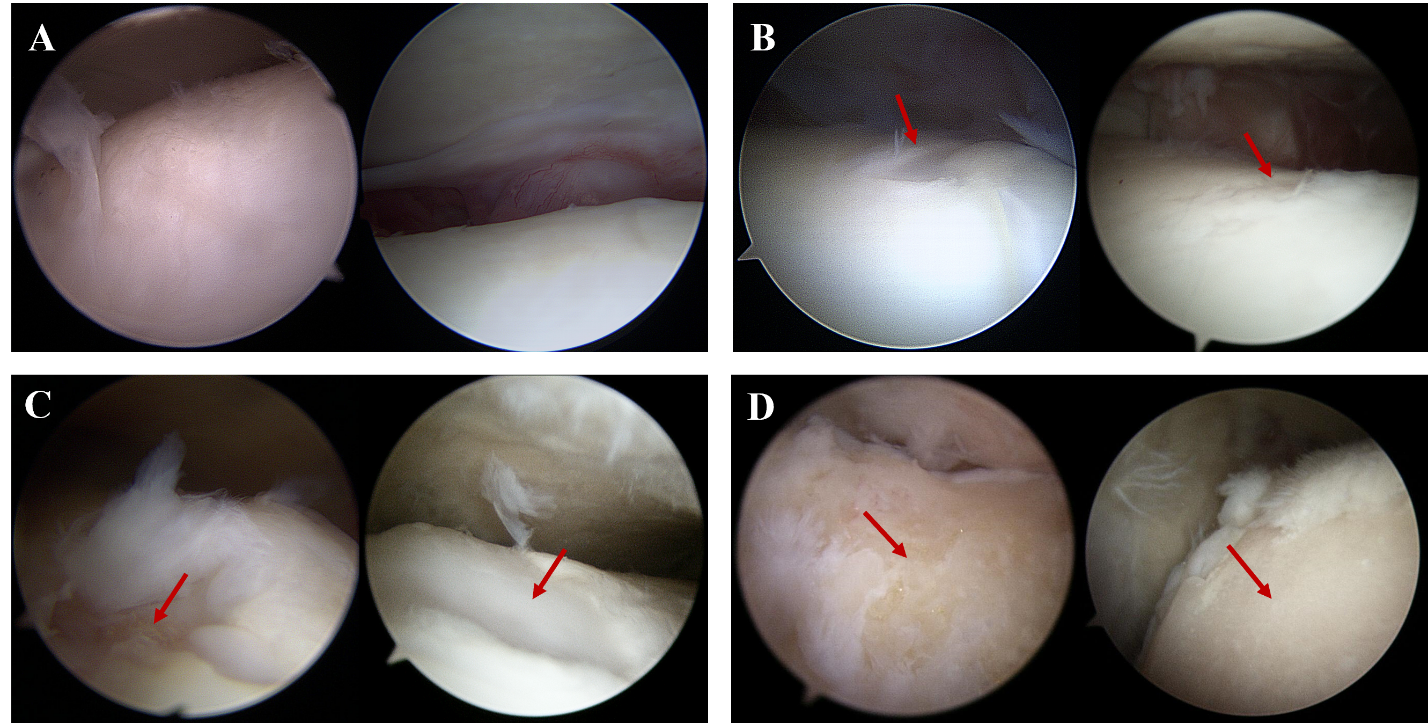
**

**Supplementary Fig. S1** Intraoperative arthroscopic images depict Outerbridge classification of cartilage degeneration (arrow) at implant removal surgery following ankle fracture: (**A**) grade I, (**B**) grade II, (**C**) grade III, (**D**) grade IV.


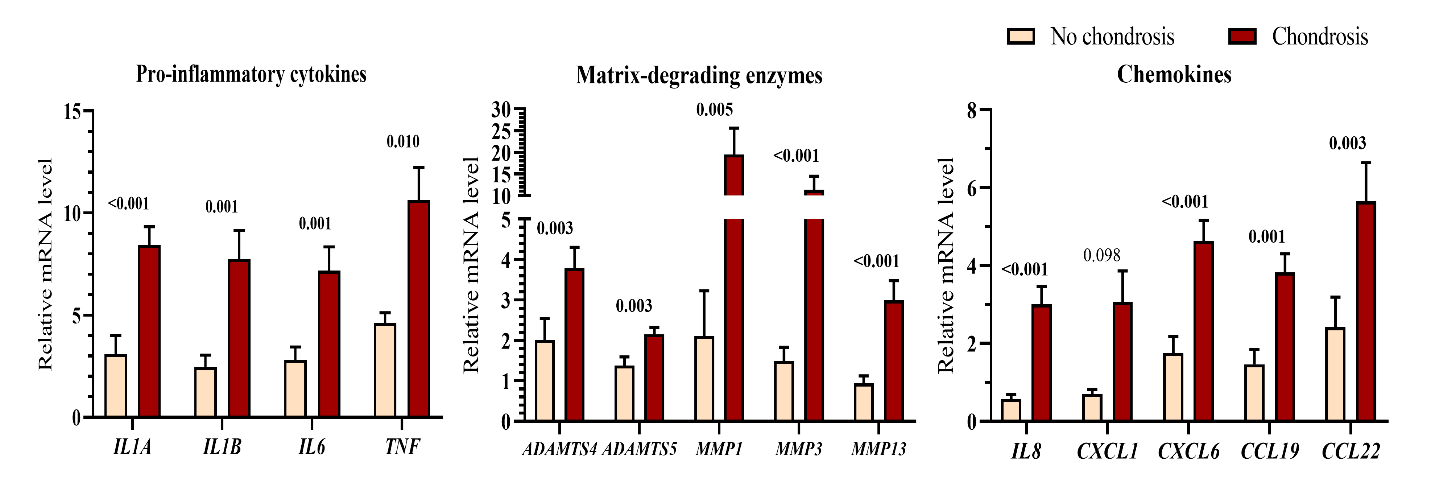


**Supplementary Fig. S2** Normalized mRNA expression levels of selected osteoarthritis-related genes in intra-articular fibrous scar tissue vary by the presence of chondrosis as defined by arthroscopy. No chondrosis group: Outerbridge I; chondrosis group: Outerbridge II-IV. The *p-*values are indicated above each bar pair. Data were analyzed by the Mann-Whitney U test.

**
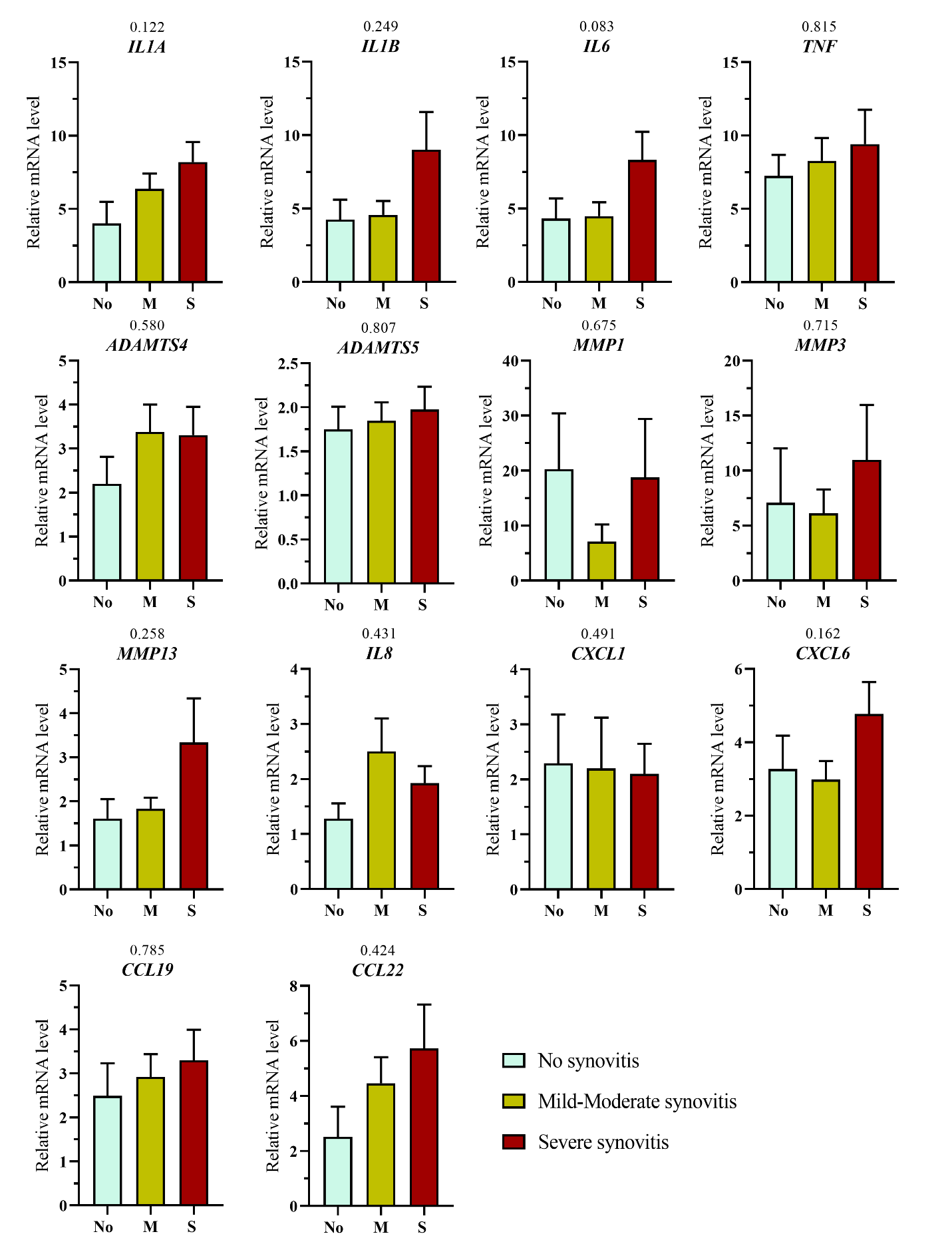
**

**Supplementary Fig. S3** Normalized mRNA expression of pro-inflammatory cytokines (*IL1A, IL1B, IL6,* and *TNF*), matrix-degrading enzymes (*ADAMTS4, ADAMTS5, MMP1, MMP3,* and *MMP13*), and chemokines (*IL8, CXCL1, CXCL6, CCL19,* and *CCL22*) in intra-articular fibrous scar tissue taken from fractured ankles classified intraoperatively according second-look arthroscopic findings of synovitis (Group No: No synovitis, group M: Mild-moderate synovitis, group S: Severe synovitis). The p-value is shown above the gene name. Data were analyzed by the Kruskal-Wallis test. Multiple comparisons were not performed because the overall test did not show significant differences between samples.
